# Employment of Independently Billing Advanced Practice Clinicians

**DOI:** 10.1001/jamahealthforum.2025.3903

**Published:** 2025-09-12

**Authors:** Parth K. Modi, Max J. Hyman, Samuel R. Kaufman, Chad Ellimoottil, Vahakn B. Shahinian, Brent K. Hollenbeck

**Affiliations:** 1 Section of Urology, Department of Surgery, University of Chicago, Chicago, Illinois; 2 The Center for Health and the Social Sciences, University of Chicago, Chicago, Illinois; 3 Dow Division of Health Services Research, Department of Urology, University of Michigan, Ann Arbor

## Abstract

This cross-sectional study uses Medicare Data on Provider Practice and Specialty files from 2008 to 2021 to evaluate changes in rates of employment of advanced practice clinicians.

## Introduction

The American Association of Medical Colleges projects a physician shortage in the US across disciplines that threatens access to primary and specialty care.^1^ With the aging population, the demand for health care will continue to increase.^2^ One solution to address the imbalance between physician supply and health care demand is the deployment of advanced practice registered nurses and physician assistants, collectively referred to as advanced practice clinicians (APCs). Depending on training background and state regulations, APCs function independently or under supervision of a physician and are a growing part of the workforce.^3,4^ Although APCs have historically been thought of as a means to expand access to primary care,^3^ the extent to which they are now being integrated into physician groups across specialties is unknown.

## Methods

This cross-sectional study was limited to APCs employed by single-specialty physician groups because the specialty focus for those employed by hospitals or by multispecialty groups is unclear. Physician groups were identified for all specialties with the Medicare Data on Provider Practice and Specialty files from 2008 to 2021. Single-specialty groups were defined as those in which a majority of physicians had the same provider specialty code. More than 90% of these groups were composed of only physicians of a single specialty. APCs were identified using specialty codes and associated with a physician group by tax identification number.^5^ To understand their deployment across specialties, physician groups were collated into broad categories of medical practice (primary care, medical specialty, surgical specialty, hospital-based specialty, obstetrics and gynecology, and psychiatry) as documented in the eTable in Supplement 1.^5^

The University of Chicago Institutional Review Board deemed this study exempt from review and waived the need for informed consent because no patient data were used. The study followed the STROBE reporting guideline.

The number of APCs and physicians employed by physician groups overall and by medical practice category was measured from 2008 through 2021. Linear regression was used to assess for the change over time in the proportion of the workforce comprising APCs overall and by practice category. Using a similar approach, the change over time in the proportion of APCs identified as advanced practice registered nurses employed by these groups was measured. Statistical analysis was performed from March 2025 to June 2025, using Stata, version 18 (StataCorp LLC); 2-sided *P* < .05 indicated statistical significance.

## Results

**Figure 1.**
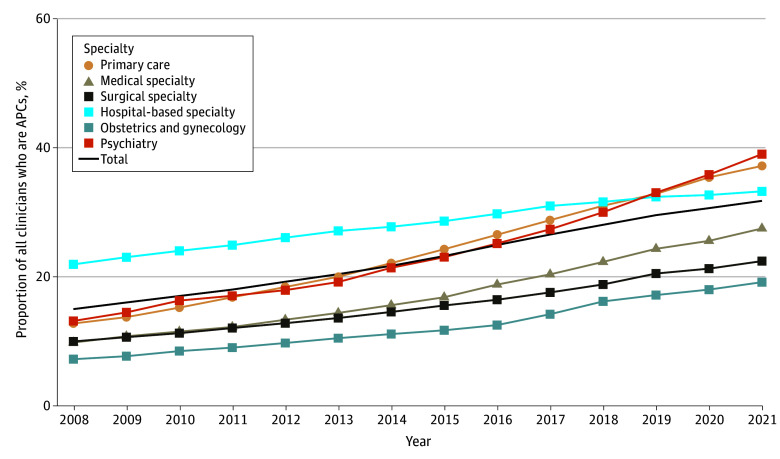
Growth in Employment of Advanced Practice Clinicians (APCs) by Medical Practice Category, 2008 to 2021 Linear regression was used to assess the change over time in the proportion of the workforce comprising APCs overall and by practice category. The data shown represent the proportion of clinicians (ie, APCs vs non-APCs). Overall *P* for trend <.01 (and for each category). Regression coefficients were as follows: hospital-based specialty, 0.009 (95% CI, 0.008-0.010), *P* < .001; medical specialty, 0.014 (95% CI, 0.012-0.015), *P* < .001; obstetrics and gynecology, 0.009 (95% CI, 0.008-0.010), *P* < .001; primary care, 0.019 (95% CI, 0.018-0.021), *P* < .001; psychiatry, 0.019 (95% CI, 0.017-0.022), *P* < .001; surgical specialty, 0.010 (95% CI, 0.009-0.011), *P* < .001. The black solid line represents all APCs and physicians who billed Medicare Part B.

**Figure 2.**
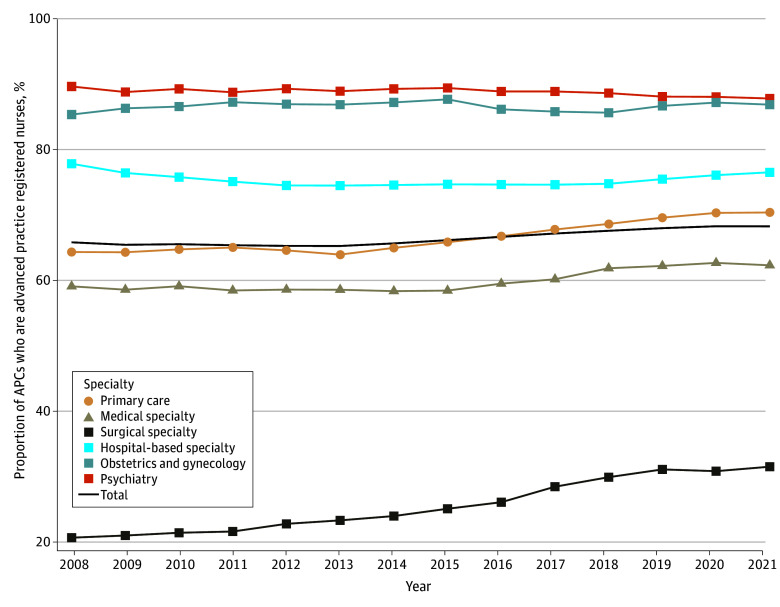
Growth in Employment of Advanced Practice Clinicians (APCs) Who Are Advanced Practice Registered Nurses by Medical Practice Category, 2008 to 2021 Linear regression was used to assess the change over time in the proportion of the APC workforce comprising advanced practice registered nurses overall and by practice category. Overall *P* for trend <.001. Regression coefficients were as follows: hospital-based specialty, –0.001 (95% CI, –0.002 to 0.001), *P* = .55; medical specialty, 0.003 (95% CI, 0.002 to 0.005), *P* < .001; obstetrics and gynecology, 0.0002 (95% CI, –0.001 to 0.001), *P* = .56; primary care, 0.005 (95% CI, 0.004-0.006), *P* < .001; psychiatry, –0.001 (95% CI, –0.002 to –0.0005), *P* = .001; surgical specialty, 0.01 (95% CI, 0.008-0.01), *P* < .001; overall, 0.003 (95% CI, 0.002-0.003), *P* < .001. The black solid line represents all APCs who billed Medicare Part B.

The total number of independently billing APCs increased from 95 241 in 2008 to 317 466 in 2021. As shown in Figure 1, the overall proportion of APCs in the workforce increased from 15.1% in 2008 to 31.8% in 2021 (*P* for trend <.001). APCs as a percentage of the workforce increased in all medical practice categories, ranging from 19.2% in obstetrics and gynecology to 39.1% in psychiatry. As shown in Figure 2, a majority of APCs were advanced practice registered nurses in all categories except for surgical specialties. However, the percentage of advanced practice registered nurses in surgical specialty groups increased significantly from 20.5% in 2008 to 31.4% in 2021 (*P* for trend <.001).

## Discussion

Independently billing APCs, predominantly composed of advanced practice registered nurses, represent a growing part of the health care workforce across specialties. These data reaffirm the shifting composition of the clinical workforce and highlight the need for continued research about the roles, specialty-specific training, quality, and cost-effectiveness of APCs. An important limitation of this study is the inability to identify (1) the specialty of APCs in multispecialty practices and (2) APCs who do not directly bill Medicare, including those in states that do not allow independent practice.^6^ This limitation notwithstanding, this study provides an important overview of the growth in APC employment across specialties over the last decade and the diverse fields in which over 300 000 APCs now provide independent care to patients receiving Medicare.

## Notes

Supplement 1. **eTable.** Practice Specialty and Type Groupings of Medicare Data on Provider Practice and Specialty (MD-PPAS) Specialties

Supplement 2.
Data Sharing Statement
