# Supplementary material for: Employment of Independently Billing Advanced Practice Clinicians
Source: JAMA Health Forum. 2025 Sep 12;6(9):e253903. doi: 10.1001/jamahealthforum.2025.3903 (PMC12432633; doi:10.1001/jamahealthforum.2025.3903)
Supplement: Supplement 1. — eTable. Practice Specialty and Type Groupings of Medicare Data on Provider Practice and Specialty (MD-PPAS) Specialties [file jamahealthforum-e253903-s001.pdf]

Supplemental Online Content

Modi PK, Hyman MJ, Kaufman SR, Ellimoottil C, Shahinian VB, Hollenbeck BK. Employment of independently billing advanced practice clinicians. *JAMA Health Forum*. Published online September 12, 2025 doi:10.1001/jamahealthforum.2025.3903

eTable. Practice Specialty and Type Groupings of Medicare Data on Provider Practice and Specialty (MD-PPAS) Specialties

This supplemental material has been provided by the authors to give readers additional information about their work.

© 2025 Modi P et al. *JAMA Health Forum*.

eTable. Practice Specialty and Type Groupings of Medicare Data on Provider Practice and Specialty (MD-PPAS) Specialties

MD-PPAS Specialty	Practice Specialty	Broad Practice Type
Family practice	General Internal Medicine	Primary Care
General practice	General Internal Medicine	Primary Care
Internal medicine	General Internal Medicine	Primary Care
Osteopathic manipulative medicine	General Internal Medicine	Primary Care
Preventative medicine	General Internal Medicine	Primary Care
Undersea and hyperbaric medicine	General Internal Medicine	Primary Care
Geriatric medicine	Geriatrics	Primary Care
Hospice and palliative care	Palliative Care	Primary Care
Addiction medicine	Addiction Medicine	Medical Specialty
Allergy/Immunology	Allergy and Immunology	Medical Specialty
Adult congenital heart disease	Cardiology	Medical Specialty
Advanced heart failure and transplant cardiology	Cardiology	Medical Specialty
Cardiac electrophysiology	Cardiology	Medical Specialty
Cardiovascular disease (cardiology)	Cardiology	Medical Specialty
Interventional cardiology	Cardiology	Medical Specialty
Dermatology	Dermatology	Medical Specialty
Micrographic dermatologic surgery	Dermatology	Medical Specialty
Endocrinology	Endocrinology	Medical Specialty
Gastroenterology	Gastroenterology	Medical Specialty
Hematology	Hematology and Oncology	Medical Specialty
Hematology/Oncology	Hematology and Oncology	Medical Specialty
Hematopoietic cell transplantation and cellular therapy	Hematology and Oncology	Medical Specialty
Medical oncology	Hematology and Oncology	Medical Specialty
Infectious disease	Infectious Disease	Medical Specialty
Medical genetics and genomics	Medical Genetics	Medical Specialty
Nephrology	Nephrology	Medical Specialty
Neurology	Neurology	Medical Specialty
Pulmonary disease	Pulmonology	Medical Specialty
Sleep medicine	Pulmonology	Medical Specialty
Rheumatology	Rheumatology	Medical Specialty
Medical toxicology	Toxicology	Medical Specialty
Cardiac surgery	Cardiac Surgery	Surgical Specialty
Colorectal surgery	Colorectal Surgery	Surgical Specialty
General surgery	General Surgery	Surgical Specialty

© 2025 Modi P et al. *JAMA Health Forum*.

Neurosurgery	Neurosurgery	Surgical Specialty
Ophthalmology	Ophthalmology	Surgical Specialty
Orthopedic Surgery	Orthopedic Surgery	Surgical Specialty
Otolaryngology	Otolaryngology	Surgical Specialty
Hand surgery	Plastic Surgery	Surgical Specialty
Plastic and reconstructive surgery	Plastic Surgery	Surgical Specialty
Surgical oncology	Surgical Oncology	Surgical Specialty
Thoracic surgery	Thoracic Surgery	Surgical Specialty
Urology	Urology	Surgical Specialty
Vascular Surgery	Vascular Surgery	Surgical Specialty
Peripheral vascular disease	Vascular Surgery	Surgical Specialty
Anesthesiology	Anesthesiology	Hospital-Based Specialty
Interventional pain management	Anesthesiology	Hospital-Based Specialty
Pain management	Anesthesiology	Hospital-Based Specialty
Critical care (intensivists)	Critical Care	Hospital-Based Specialty
Emergency medicine	Emergency Medicine	Hospital-Based Specialty
Hospitalist	Hospital Medicine	Hospital-Based Specialty
Pathology	Pathology	Hospital-Based Specialty
Physical medicine and rehabilitation	Physical Medicine and Rehabilitation	Hospital-Based Specialty
Sports medicine	Physical Medicine and Rehabilitation	Hospital-Based Specialty
Radiation oncology	Radiation Oncology	Hospital-Based Specialty
Diagnostic radiology	Radiology	Hospital-Based Specialty
Interventional radiology	Radiology	Hospital-Based Specialty
Nuclear medicine	Radiology	Hospital-Based Specialty
Gynecological/Oncology	Obstetrics and Gynecology	Obstetrics and Gynecology
Obstetrics/Gynecology	Obstetrics and Gynecology	Obstetrics and Gynecology
Geriatric psychiatry	Psychiatry	Psychiatry
Neuropsychiatry	Psychiatry	Psychiatry
Psychiatry	Psychiatry	Psychiatry

© 2025 Modi P et al. *JAMA Health Forum*.
