# Supplementary material for: Employment of Independently Billing Advanced Practice Clinicians
Source: JAMA Health Forum. 2025 Sep 12;6(9):e253903. doi: 10.1001/jamahealthforum.2025.3903 (PMC12432633; doi:10.1001/jamahealthforum.2025.3903)
Supplement: Supplement 2. — Data Sharing Statement [file jamahealthforum-e253903-s002.pdf]

Data Sharing Statement

Modi. Employment of Independently Billing Advanced Practice Clinicians. *JAMA Health Forum*. Published September 12, 2025. doi:10.1001/jamahealthforum.2025.3903

Data
Data available: No

Additional Information
Explanation for why data not available: Data dictionary publicly available. Data itself used through a DUA with CMS.
